# Transanal irrigation in pediatric bowel dysfunction: a prospective study on clinical outcomes and quality of life

**DOI:** 10.1007/s00383-025-06279-1

**Published:** 2025-12-29

**Authors:** Marco Di Mitri, Annalisa Di Carmine, Simone D’Antonio, Francesca Ruspi, Cristian Bisanti, Edoardo Collautti, Sara Maria Cravano, Michele Libri, Riccardo Coletta, Roberto Lo Piccolo, Tommaso Gargano, Enrico Ciardini, Mario Lima

**Affiliations:** 1 Pediatric Surgery Department, IRCCS Meyer, Florence, Italy; 2 Faculty of Medicine, Alma Mater Studiorum—Università degli Studi di Bologna, Bologna, Italy; 3 Pediatric Surgery Department, IRCCS Sant’Orsola-Malpighi, Alma Mater Studiorum, Università degli Studi di Bologna, Bologna, Italy; 4 Department of Neurofarba, University of Florence, 50121 Firenze, Italy

## Abstract

**Objectives:**

Transanal irrigation (TAI) is an emerging minimally invasive therapy for children with severe bowel dysfunction, including fecal incontinence and refractory constipation. The aim of this study was to evaluate the impact of TAI on bowel habits, medication use, quality of life, and caregiver burden in a pediatric population.

**Methods:**

A prospective observational study was conducted on 20 pediatric patients with fecal incontinence. A structured questionnaire assessing bowel function and quality of life was administered at baseline and after 60 days of TAI. Outcomes were compared using Fisher’s exact and Wilcoxon signed-rank tests.

**Results:**

Significant improvements were observed in bowel care time (*p* < 0.01), frequency of soiling (*p* < 0.01), fecal incontinence episodes (*p* < 0.01), and dependence on laxatives or enemas (*p* < 0.01). Social participation and emotional well-being also improved. Most patients reported better autonomy and reduced discomfort related to bowel management.

**Conclusions:**

TAI is an effective strategy to improve continence, reduce the burden of bowel care, and enhance quality of life in children with refractory bowel dysfunction.

## Introduction

Bowel dysfunction is a common and complex problem in pediatric patients [1]. This condition is frequently observed in patients affected by congenital or acquired pathologies such as anorectal malformations (ARM), Hirschsprung’s disease (HD), neurogenic bowel dysfunction (NBD), and idiopathic megarectum, resulting in chronic constipation and/or fecal incontinence [2, 3]. These problems significantly have an impact on the patient’s physical health, psychosocial development, and patient’s family or caregivers. Children affected often experience difficulties in achieving continence despite surgical correction or optimal medical management, and many of them remain dependent on intensive bowel care routines until late adolescence [4]. 

The appropriate treatment of these disorders poses a multifaceted challenge. Standard therapeutic approaches typically include dietary modifications, the use of oral laxatives, rectal enemas, and stool softeners [5]. In severe cases, patients may require mechanical assistance or surgical procedure such as antegrade continence enemas (ACE) or colostomy [6]. However, these options may be invasive and associated with complications or reduced patient’s compliance [7]. 

In the last years, there is a growing interest in less invasive strategies that can improve bowel control while promoting autonomy and preserving quality of life.

Transanal irrigation (TAI) has emerged as a valuable alternative in the treatment of pediatric bowel dysfunction. This technique involves the introduction of a controlled volume of water through a catheter into the rectum, aiming to stimulate colonic emptying and preventing fecal retention. TAI can reduce fecal incontinence, alleviate constipation and improve bowel regularity. It may also delay or prevent the need for surgical options in selected patients [8]. 

While TAI is a well-defined and tested procedure in adult populations with neurologic bowel disorders (spinal cord injury or multiple sclerosis), its use in children is relatively recent and supported by limited but promising evidence. In the pediatric setting, TAI is not only a therapeutic intervention but also a tool for fostering independence, self-esteem, and encouraging the child’s participation in daily activities [9]. Despite its potential, few studies have comprehensively assessed the real-world impact of TAI on the lifestyle of children and adolescents, or on the impact experienced by their caregivers [10]. This prospective observational study aims to evaluate the effects of TAI in a cohort of pediatric patients with severe bowel dysfunction. Our objectives were to explore changes in bowel habits, time and effort dedicated to bowel care, use of medications, and continence status. In addition, we collected patient-reported outcomes concerning quality of life, social participation, emotional well-being, and caregiver involvement before and after 60 days from the beginning of the TAI.

## Methods

### Ethics statement

This prospective observational study was conducted at the Pediatric Surgery Unit of the IRCCS Azienda Ospedaliero-Universitaria di Bologna (AOUBO) in accordance with the principles of the Declaration of Helsinki. The study protocol was reviewed and approved by the Ethics Committee of the IRCCS Azienda Ospedaliero-Universitaria di Bologna (protocol code: CHPED-TAI). Written informed consent was obtained from the patients’ parents or legal guardians prior to participation.

### Study design

**Fig. 1:**
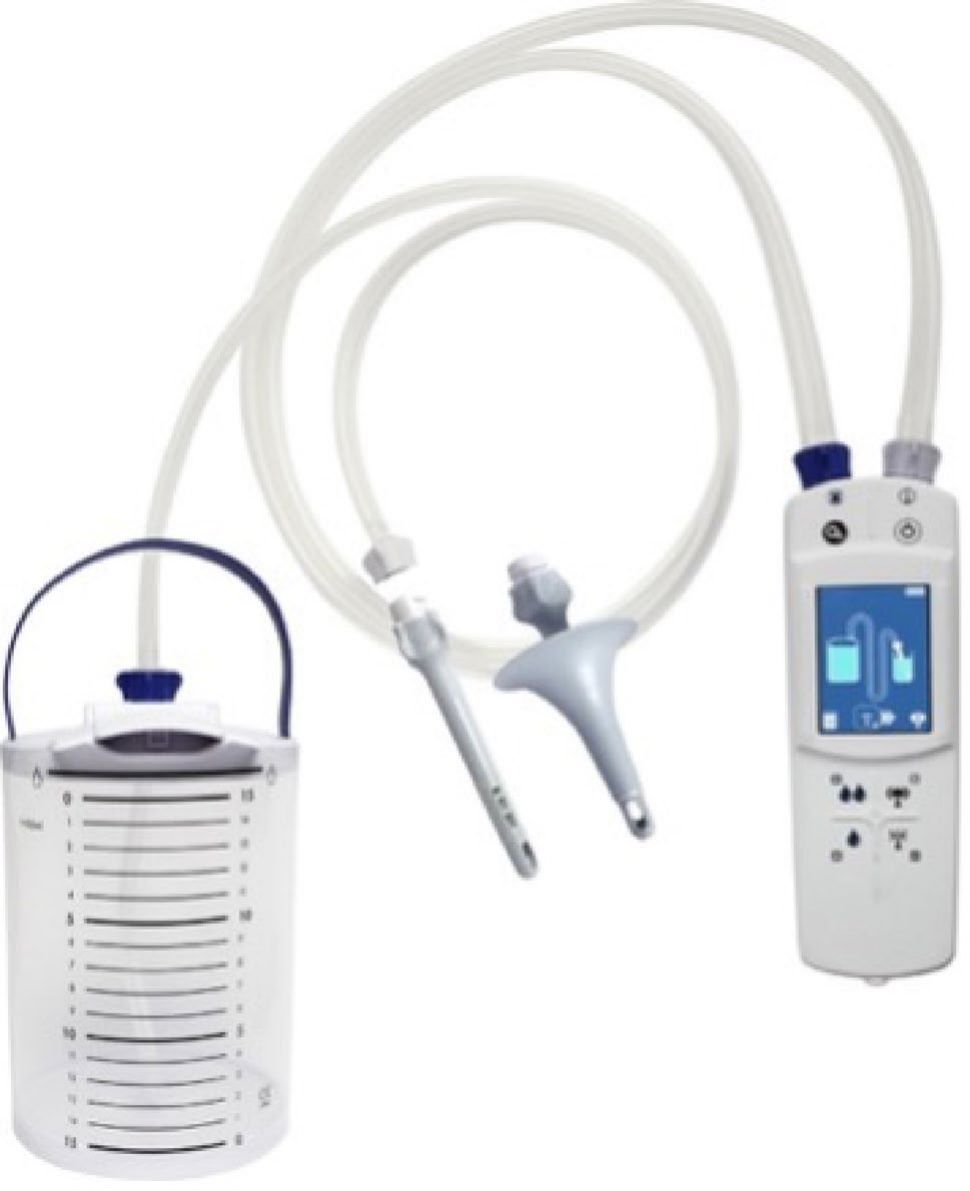
Navina Smart^®^ (Wellspect HealthCare) system for transanal irrigation (TAI), used in this study for the management of pediatric bowel dysfunction

The study included pediatric patients with a diagnosis of fecal incontinence who were scheduled to begin transanal irrigation (TAI) therapy using NAVINA Smart - Wellspect HealthCare (Fig. 1). The study enrollment period extended from December 1, 2023, to January 1, 2025. We designed a single-arm before–after (pre–post) study in which all patients initially receiving treatment (A) were subsequently switched to treatment (B) (TAI). Outcomes measured after the switch were compared with those observed during treatment A.

### Participants

Eligible patients were identified during routine outpatient visits. Inclusion criteria were:

- Age between 2 and 18 years at the time of the enrollment.
- Diagnosis of primary or secondary fecal incontinence.

### Intervention and data collection

**Fig. 2:**
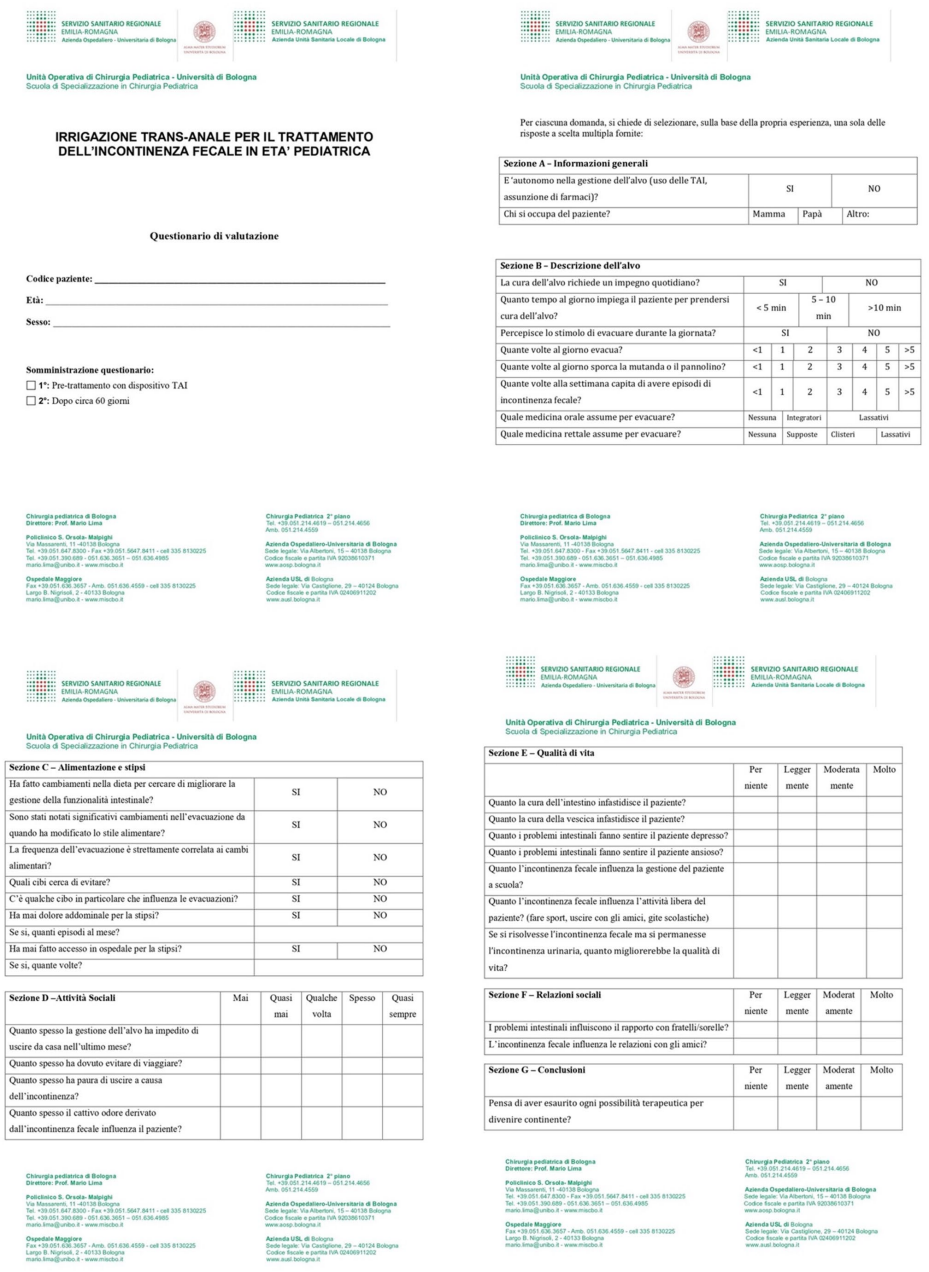
The questionnaire administered to the parents of the patients

All enrolled patients started treatment with TAI as part of their clinical care. A structured questionnaire (Fig. 2) was administered at two timepoints:

1. Baseline: during the outpatient visit prior to starting TAI.
2. Follow-up: approximately 60 days after starting TAI, during a scheduled follow-up visit.

The questionnaire administered in this study was adapted from the Fecal Incontinence and Constipation Quality of Life (FIC-QOL) tool, originally developed and validated for children with spina bifida [11]. 

The questionnaire was designed to be completed jointly by the patient and their caregiver, with the support of the attending physician when needed. It was divided in seven sections:

- Sections A, B, and C: bowel management, symptoms, and medication use.
- Sections D, E, F, and G: quality of life, emotional impact, and social functioning.

Clinical data, including demographic, diagnoses and comorbidities were retrieved from electronic medical records and outpatient visit reports.

### Transanal irrigation procedure

The procedure is usually performed with the patient seated on the toilet, or in younger children lying on their side. The irrigation system is prepared by filling the water reservoir with lukewarm water (around 35–38 °C) and connecting it to the tubing and rectal catheter or cone. The rectal device is well lubricated and gently inserted into the anus. In systems with a balloon catheter, the balloon is then inflated to secure the device and prevent leakage.

Once the device is in place, water is instilled slowly into the rectum and distal colon. The amount of water depends on the age and size of the patient: in children, volumes typically range from 100 to 400 ml. The instillation should proceed at a controlled pace to minimize discomfort, and it can be interrupted if the child experiences pain or resistance.

After the desired volume has been delivered, the catheter or cone is carefully removed (deflating the balloon first, if present). The patient then remains seated on the toilet to allow evacuation of the infused water together with stool. This usually occurs in several waves over a period of 20 to 45 min.

At the end of the procedure, the disposable parts of the system are discarded, and the reusable components are cleaned according to the manufacturer’s instructions. Regular repetition of the procedure—daily or every other day depending on the clinical indication—helps to maintain predictable bowel emptying and improve continence. Parents were instructed to use age-appropriate volumes of lukewarm tap water (100–400 mL) and to report any symptoms of abdominal distension, lethargy, or excessive thirst. No clinical signs of electrolyte imbalance or water intoxication were detected during the study period.

### Statistics

Descriptive statistics were used to summarize patient demographics and baseline clinical characteristics. Categorical variables were presented as absolute numbers and percentages, and continuous variables were reported as means and standard deviations (SD).

To assess the impact of TAI, we compared pre- and post-treatment questionnaire responses. Paired data were analyzed using:

- The Wilcoxon signed-rank test for ordinal or non-normally distributed continuous variables.
- The Fisher’s exact test for paired categorical variables with binary outcomes.

All statistical analyses were conducted using Statistical Package for the Social Sciences - SPSS. A p-value of < 0.05 was considered statistically significant.

## Results

### Patient characteristics

We included 20 pediatric patients, 14 males and 6 females, with a mean age of 12.5 years (± 5 SD). The underlying diagnoses were anorectal malformation (*n* = 12), megarectum (*n* = 3), neurogenic bowel (*n* = 3), and Hirschsprung’s disease (*n* = 2). Three patients (aged 9, 16, and 17 years) were able to independently perform transanal irrigations. In the remaining 17 cases, the procedure was carried out by a caregiver: in 15 cases by the mother and in 2 by the father (Table 1). The low proportion of independent users reflected the young age and complexity of bowel dysfunction in our cohort. Most children required caregiver support due to anatomical/functional limitations and familiarity with the procedure, as similarly reported in previous pediatric TAI studies.

**Table 1:** Patients characteristics

Patients data (*n* = 20)	
Age, years	12.5 ± 5.0 (mean ± SD)
Sex, male/female	14 (70%)/6 (30%)
Diagnosis	ARM: 12 (60%) Megarectum: 3 (15%) Neurogenic bowel: 3 (15%) Hirschsprung disease: 2 (10%)
Independent TAI users	3 (ages 9, 16, 17 years)
Caregiver-performed TAI	Mother: 15 (75%) Father: 2 (10%)

### Daily effort required for bowel management

Before starting TAI, 13 patients reported that bowel care required daily effort. This number decreased to 9 after treatment, although the difference did not reach statistical significance (*p* = 0.341).

### Time spent daily on bowel care

Before starting TAI, 16 patients required more than 10 min per day for bowel care, 3 needed 5–10 min, and 1 patient less than 5 min. After treatment, 11 required more than 10 min, 2 between 5 and 10 min, and 7 less than 5 min. This improvement was statistically significant (*p* < 0.01).

### Perception of the urgency to defecate

Ten patients reported perceiving the urgency to defecate before using TAI. After treatment, this number increased to 16, indicating a positive trend, although not statistically significant (*p* = 0.09).

### Frequency of daily evacuations

Before treatment, 4 patients evacuated less than once per day, and 6 had three or more evacuations daily. After treatment, 13 patients reported one regular daily evacuation, and none had more than three. This change was statistically significant (*p* = 0.01).

### Frequency of daily soiling episodes

Ten patients experienced three or more daily soiling episodes prior to treatment, including 4 with more than five. After experimenting TAI, 16 patients reported fewer than one soiling episode per day, and none exceeded two episodes. This reduction was highly statistically significant (*p* < 0.01).

### Weekly fecal incontinence episodes

Before starting TAI, 13 patients experienced two or more weekly episodes of fecal incontinence, with 5 reporting more than five episodes. After treatment, 15 patients had fewer than one episode per week, and none exceeded two. This improvement was also highly statistically significant (*p* < 0.01).

### Use of oral medications

All patients used oral laxatives before starting TAI. After treatment, only 6 continued using laxatives and one switched to fiber supplements. This reduction was highly significant (*p* < 0.01).

### Use of rectal medications

Prior to TAI, rectal enemas were used by all 20 patients. After treatment, none of the patients required enemas, showing an extremely significant change (*p* < 0.01).

### Dietary modifications and bowel function

At the time of enrollment, 10 out of 20 patients had previously modified their diet to improve bowel function. However, only 5 of these patients reported that dietary changes had led to a significant improvement in bowel evacuation, while 15 did not perceive any notable effect.

### Abdominal pain related to constipation

Before starting TAI, 8 out of 20 patients reported experiencing abdominal pain related to constipation. Following treatment, this number decreased to 2. Although the reduction did not reach statistical significance, it showed a strong trend toward clinical improvement (*p* = 0.065).

### Hospital admissions due to constipation

Before starting TAI, 7 out of 20 patients had experienced hospital admissions related to constipation. After treatment, this number decreased to 3. Although the reduction suggests clinical improvement, it did not reach statistical significance (*p* = 0.273).

### Impact on leaving the house

Before treatment, several patients reported limitations in leaving the house going somewhere, experiencing these situations “sometimes” or even “often.” Following the introduction of TAI, most patients reported “never” referring to the experiencing these limitations/have a feeling of being restricted. This improvement was statistically significant (*p* < 0.01).

### Impact on traveling

The frequency with which patients avoided traveling due to bowel management significantly decreased following TAI. Prior to therapy, several patients reported avoiding travel “sometimes” or “often.” After the introduction of TAI, most patients answered “never,” indicating a statistically significant improvement (*p* < 0.01).

### Fear of leaving the house due to incontinence

The frequency with which patients reported being afraid to leave the house because of fecal incontinence significantly decreased after starting TAI. Before treatment, several patients reported this fear “sometimes,” “often,” or “almost always.” After treatment, most responded “never” or “rarely,” indicating a statistically significant improvement (*p* = 0.01).

### Impact of odor related to fecal incontinence

The influence of fecal odor on patients’ daily lives significantly decreased after the introduction of TAI. Prior to treatment, many patients reported being affected by odor “sometimes” or “often.” Following treatment, the majority answered “never” or “rarely,” reflecting a statistically significant improvement (*p* < 0.01).

### Discomfort caused by bowel care

The level of discomfort caused by bowel care significantly decreased following the introduction of TAI. Prior to treatment, many patients reported feeling “moderately” or “very” bothered. After starting TAI, the majority reported little or no discomfort. This improvement was statistically significant (*p* < 0.01).

### Emotional impact–depression

Several patients reported feeling emotionally affected by their intestinal issues before starting TAI, with some describing moderate to high levels of depression. Following treatment, most patients reported little to no depressive feelings related to their bowel condition. Although this trend suggested clinical improvement, the difference did not reach statistical significance (*p* = 0.132).

### Emotional impact–anxiety

Before starting TAI, several patients reported feeling moderate to high levels of anxiety due to intestinal issues. After treatment, most reported little or no anxiety. While the trend indicated improvement, the difference did not reach statistical significance (*p* = 0.102).

### Impact of fecal incontinence on school life

The influence of fecal incontinence on school life significantly decreased following TAI. Prior to therapy, several patients reported that incontinence had a moderate or severe impact on their ability to attend and participate in school. After treatment, most described this impact as minimal or nonexistent. This improvement was statistically significant (*p* = 0.046).

### Impact of fecal incontinence on free-time activities

The impact of fecal incontinence on free-time activities—including sports, social outings, and school trips—significantly decreased after the introduction of TAI. Before treatment, several patients reported moderate to severe limitations. After treatment, most described little to no interference. This improvement was statistically significant (*p* = 0.01).

### Relationship with siblings

The reported influence of intestinal problems on relationships with siblings remained low both before and after TAI. Most patients indicated that these issues did not affect their sibling relationships. The difference was not statistically significant (*p* = 0.317, Wilcoxon signed-rank test).

### Relationship with friends

Several patients initially reported that fecal incontinence affected their relationships with peers, ranging from mild to moderate interference. After TAI, many of these limitations improved, though the difference was not statistically significant (*p* = 0.271). (Table 2)

**Table 2:** Summary of patient outcomes before and after TAI

Outcome measure	Pre-TAI	Post-TAI	*P*-value
*Section B–Bowel management*			
Daily bowel care perceived as effortful (n)	13	9	0.341
Time spent > 10 min/day on bowel care (n)	16	11	< 0.01
Urge to defecate perceived (n)	10	16	0.09
1 regular evacuation/day (n)	7	13	0.01
≥ 3 daily soiling episodes (n)	10	0	< 0.01
≥ 2 weekly incontinence episodes (n)	13	0	< 0.01
Oral laxatives use (n)	20	6	< 0.01
Rectal enemas use (n)	20	0	< 0.01
*Section C–Diet and constipation*			
Abdominal pain due to constipation (n)	8	2	0.065
Hospital admissions for constipation (n)	7	3	0.273
*Section D–Social activity*			
Restricted from leaving home (any frequency > 0)	13	4	< 0.01
Avoided travel (any frequency > 0)	13	4	< 0.01
Fear of leaving due to incontinence (any frequency > 0)	15	5	0.01
Odor-related impact (any frequency > 0)	15	4	< 0.01
*Section E–Quality of Life*			
Discomfort from bowel care (moderate/severe)	13	5	< 0.01
School life affected by incontinence (moderate/severe)	8	3	0.04
Free-time activities affected (moderate/severe)	12	4	0.01
*Section F–Social relationships*			
Siblings relationship affected (any degree)	3	2	0.317
Friend relationships affected (moderate/severe)	13	6	0.271
*Section G–Treatment perception*			
Mean perceived therapeutic exhaustion (0–3 scale)	1.06 ± 0.97	1.33 ± 0.97	0.290

## Discussion

This prospective observational study demonstrates that TAI provides significant clinical and functional benefits in a cohort of pediatric patients suffering from severe bowel dysfunction due to a variety of underlying conditions, including anorectal malformations, Hirschsprung’s disease, megarectum, and neurogenic bowel.

Our results confirm previous studies published in the literature suggesting that TAI a valuable therapeutic tool in pediatric patients for improving bowel continence, reducing caregiver burden, and enhancing overall quality of life [12, 13]. 

We observed a statistically significant reduction in the time required for daily bowel management, the frequency of soiling episodes and weekly fecal incontinence. These results are consistent with the outcomes reported by Velde et al. and Christensen et al., who described improved fecal continence and reduced time spent on bowel care in children using TAI systems [14, 15]. One of the most relevant findings is the discontinuation of enemas and significant reduction in oral laxatives after TAI initiation. This has been previously reported in the pediatric literature and confirms TAI’s role in replacing more invasive or pharmacologic approaches with a better-tolerated and effective regimen. The complete cessation of enemas in our cohort (from 100% to 0%) is particularly striking and supports its role as a non-pharmacological alternative [10, 16]. Functionally, our cohort showed a shift toward regular daily evacuations and increased perception of rectal urgency, reflecting a more physiological bowel pattern. These outcomes are comparable to those observed in the pediatric TAI series by Jorgensen et al. and Baaleman et al., which reported similar changes in bowel rhythm and a trend toward improved awareness and continence [10, 17]. On the psychosocial front, our data show statistically significant improvements in the ability to leave the house, travel, and participate in school and leisure activities. Although changes in anxiety and depression scores did not reach statistical significance, the overall trend was favorable a finding that mirrors report by Choi et al. and Bazzocchi et al., who emphasized that even modest improvements in continence can yield substantial psychosocial gains [18, 19]. These results highlight how TAI may impact not just the physical but also the emotional and social dimensions of care, which are particularly critical during childhood and adolescence. The reason why some outcomes, such as interpersonal relationships or perception of therapeutic exhaustion, did not change significantly could be due to the relatively short follow-up (60 days) or the multifactorial etiology of emotional distress in these patients. Overall, our findings support the growing body of evidence indicating that TAI is a well-tolerated, effective, and patient-centered strategy for managing chronic bowel dysfunction in children. Although adverse events are rare when appropriate volumes are used, safety monitoring remains essential. In our cohort, no clinical signs of electrolyte imbalance, water intoxication, or fluid overload were observed during the follow-up period. Notably, our study is among the few to employ a structured, multidimensional questionnaire assessing not only clinical parameters but also social integration, quality of life, and emotional well-being.

Although our cohort included children with different underlying diagnoses, this reflects the heterogeneous population typically encountered in pediatric clinical practice. The common final pathway of refractory bowel dysfunction justified their inclusion. Nevertheless, we acknowledge that heterogeneity may limit direct comparison across subgroups, and studies focusing on specific diagnoses are warranted.

This study has several limitations. First, the sample size was relatively small, which may limit the statistical power of certain analyses and the generalizability of the findings. Second, the observational design does not allow for causal inferences. Third, the follow-up period of 60 days may be insufficient to fully capture the long-term benefits or adherence to TAI. Additionally, some of the outcome measures relied on subjective patient’s point of view or caregiver reporting, which may be prone to bias.

## Conclusions

Transanal irrigation is a safe, well-tolerated, and effective therapy for improving bowel function and quality of life in children with severe bowel dysfunction. The treatment not only reduces fecal incontinence and the need for medications but also contributes to improve autonomy and psychosocial well-being. Further studies with larger cohorts and longer follow-up are needed to confirm these results and optimize patient selection and treatment protocols.

## Data Availability

No datasets were generated or analysed during the current study.
